# Adverse reactions of dimethyl sulfoxide in humans: a systematic review

**DOI:** 10.12688/f1000research.16642.2

**Published:** 2019-08-06

**Authors:** Bennedikte Kollerup Madsen, Maria Hilscher, Dennis Zetner, Jacob Rosenberg

**Affiliations:** 1 Department of Surgery, Center for Perioperative Optimization (CPO), Herlev Hospital, Herlev, 2730, Denmark

**Keywords:** Dimethyl Sulfoxide, DMSO, Adverse reactions, Toxicology

## Abstract

**Background:** Dimethyl sulfoxide (DMSO) has been used for medical treatment and as a pharmacological agent in humans since the 1960s. Today, DMSO is used mostly for cryopreservation of stem cells, treatment of interstitial cystitis, and as a penetrating vehicle for various drugs. Many adverse reactions have been described in relation to the use of DMSO, but to our knowledge, no overview of the existing literature has been made. Our aim was to conduct a systematic review describing the adverse reactions observed in humans in relation to the use of DMSO.

**Methods:** This systematic review was reported according to the PRISMA-harms (Preferred Reporting Items for Systematic reviews and Meta-Analysis) guidelines. The primary outcome was any adverse reactions occurring in humans in relation to the use of DMSO. We included all original studies that reported adverse events due to the administration of DMSO, and that had a population of five or more.

**Results:** We included a total of 109 studies. Gastrointestinal and skin reactions were the commonest reported adverse reactions to DMSO. Most reactions were transient without need for intervention. A relationship between the dose of DMSO given and the occurrence of adverse reactions was seen.

**Conclusions:** DMSO may cause a variety of adverse reactions that are mostly transient and mild. The dose of DMSO plays an important role in the occurrence of adverse reactions. DMSO seems to be safe to use in small doses.

**Registration: **PROSPERO CRD42018096117.

## Introduction

The first medical report on the use of dimethyl sulfoxide (DMSO) as a pharmacological agent was published in 1964 ^1^. A year later, the use of DMSO in humans was terminated because experimental studies had shown refractive index changes to the lens of the eye ^1, 2^. Years later, DMSO was again approved for use in humans since this side effect was only proven in animal studies ^2^. DMSO has since been used for a variety of purposes, such as treatment of musculoskeletal and dermatological diseases, cryopreservation of stem cells, treatment of interstitial cystitis, treatment of increased intracranial pressure, and many more ^3– 9^.

DMSO is a colourless liquid, which is rapidly absorbed when administered dermally or orally ^10, 11^. DMSO is used as a cryoprotectant because it decreases osmotic stress and cellular dehydration, and thereby enables stem cells to be stored for several years ^12^. DMSO is mostly excreted through the kidneys, but a small part is excreted through the lungs and liver ^10^. Part of the DMSO is transformed to the volatile metabolite dimethyl sulfide, which gives a characteristic garlic- or oyster-like smell when excreted through the lungs ^10^. DMSO may induce histamine release, which can be the reason for adverse reactions such as flushing, dyspnoea, abdominal cramps, and cardiovascular reactions ^11^.

To our knowledge, no systematic reviews have been performed on the adverse reactions of DMSO. Our aim was therefore to provide an extensive overview of the suspected adverse reactions to DMSO in humans.

## Methods

### Protocol and eligibility criteria

Our study-protocol is registered at PROSPERO (Registration number: CRD42018096117). The systematic review was performed according to PRISMA-harms (Preferred Reporting Items for Systematic Reviews and Meta-Analyses) guidelines ^13^.

No limitations were set on the date of publication. The language was restricted to English, Danish, Swedish, Norwegian, and Russian. We included all original studies that administered DMSO to humans and included five or more participants. There was no gender or age restriction. For a study to be included, the authors had to suspect that an observed adverse reaction could be caused by DMSO.

### Primary outcome

The primary outcome was any adverse reaction seen in relation to the use of DMSO in humans.

### Literature search

The search was performed in PubMed (1966-present), EMBASE (1980-present), and the Cochrane Library. The databases were last searched on February 23, 2018. Our search strategy was formulated with the help of a medical research librarian.

The search string used in PubMed was: ((dimethyl sulfoxide) OR DMSO) AND ((((((administration and dosage) OR adverse reactions) OR alternate effects) OR secondary response) OR toxicology) OR side effects)). The search was restricted to humans. The search string was adapted to EMBASE and Cochrane Library using the same search-words as abovementioned.

The search string used in EMBASE was: ((dmso or dimethyl sulfoxide) and ((side effect or toxicology or secondary response or alternate effects or alternate reactions or (administration and dosage)) and (dmso or dimethyl sulfoxide))).mp. The search was restricted to humans, articles and Medline journals were excluded.

The search string used in Cochrane was: (adverse drug events and dimethyl sulfoxide). The search was restricted to trials.

### Study selection and data extraction

Two authors (B.K.M. and D.Z.) independently screened title and abstract according to the eligibility criteria using www.covidence.org. Discrepancies were resolved by discussion. One author screened the full-text articles (B.K.M.). Russian articles were screened by an author fluent in Russian (M.H.). If M.H. was in doubt regarding inclusion of a study the results were presented to B.K.M. and then discussed until a mutual decision was made.

After the screening process was finished, all included studies were imported to an Excel sheet (Microsoft Excel 2016). Data extraction was performed by two authors (M.H. extracted from the Russian articles and B.K.M. extracted from the rest). Data extracted were: author, publication year, country, study characteristics (study design, sample size, size of comparison group if present, time to follow-up), use of DMSO (reason for use, treatment duration, administration route, dose of DMSO), and adverse reactions observed (number of persons experiencing an adverse reaction, method of assessing, and duration of adverse reaction).

### Analysis

The Newcastle-Ottawa-Scale was used to assess the risk of bias in non-randomized observational studies ^14^. Risk of bias in randomized controlled trials was assessed using the Cochrane Handbook “Risk of Bias” assessment tool ^15^. Risk of bias was assessed at the outcome level.

The primary summary measure was percentage of persons experiencing an adverse reaction, as well as the range in which a reaction occurred in the studies included. No meta-analysis and further summery measures were planned due to the expected large heterogeneity of the studies.

## Results

### Study selection

**Figure 1.**
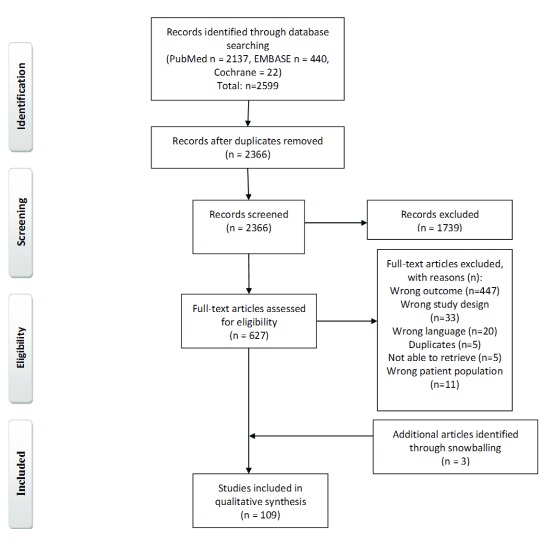
PRISMA flow diagram.

Our primary search identified 2599 studies ( Figure 1). After the evaluation process, 109 studies were included in the final review ^2, 4, 6– 9, 16– 118^.

### Gastrointestinal reactions

Gastrointestinal adverse reactions were reported in 61 studies. Of these, 10 studies were randomized controlled trials ^16, 30, 33, 55, 57, 59, 67, 79, 93, 95^, 49 were cohort studies ^2, 4, 7, 9, 18, 19, 23, 25– 27, 29, 35, 38– 43, 45, 46, 48, 50– 54, 58, 60, 66, 68, 69, 71, 73, 83, 85– 88, 90, 94, 97, 98, 101, 104, 105, 112, 113, 115, 118^, and 2 were case series ^84, 109^. Most studies reported the number of patients experiencing an adverse reaction ( Table 1). Other studies reported adverse reactions observed in relation to the number of treatments given ( Table 2).

**Table 1.** Gastrointestinal adverse reactions observed per number of patients.

Adverse reaction	Studies	Total patients, n	Patients with adverse reaction, n (%)	(%, min-max) †
**Nausea** *(overall incidence)*	[ 2, 18, 27, 33, 45, 46, 48, 53, 55, 57, 59, 60, 67, 84, 90, 93, 109, 118]	2214	257 (12)	(2–41) [ 55] - [ 48]
Intravenous administration	[ 18, 27, 33, 46, 48, 53, 59, 90, 118]	1154	199 (17)	(2–41) [ 59] - [ 48]
Transdermal application	[ 2, 45, 55, 57, 67, 93, 109]	1039	51 (5)	(2–32) [ 55] - [ 2]
>1 administration route	[ 60, 84]	21	7 (33)	(29–36) [ 84] - [ 60]
**Vomiting** *(overall incidence)*	[ 2, 18, 27, 33, 46, 48, 55, 57, 59, 118]	1611	115 (7)	(0–64) [ 55] - [ 48]
Intravenous administration	[ 18, 27, 33, 46, 48, 59, 118]	972	108 (11)	(2–64) [ 59] - [ 48]
Transdermal application	[ 2, 55, 57]	639	7 (1)	(0–6) [ 55] - [ 2]
**Nausea and vomiting** ‡	[ 7, 38, 41, 54, 66, 69, 73, 85, 87, 115]	4529	591 (13)	(0–46) [ 66] - [ 73]
**Abdominal cramps/stomach** **ache** *(overall incidence)*	[ 18, 26, 27, 39, 41, 54, 55, 59, 73, 85, 87, 93, 115]	1629	88 (5)	(1–52) [ 117] - [ 116]
Intravenous administration	[ 18, 26, 27, 39, 41, 54, 59, 73, 85, 87, 115]	1253	72 (6)	(1–52) [ 18] - [ 26]
Transdermal application	[ 55, 93]	376	16 (4)	(2–16) [ 55] - [ 93]
**Halitosis/garlic-like breath** *(overall incidence)*	[ 4, 9, 16, 19, 29, 30, 35, 42, 43, 45, 50, 52, 55, 57, 58, 66– 68, 79, 83, 85, 88, 94, 95, 97, 98, 109, 112, 113]	5782	607 (11)	(0–100) [ 30] - [ 19, 43, 45, 83, 98]
Intravenous administration	[ 16, 85, 94, 98]	239	14 (6)	(1–100) [ 85] - [ 98]
Transdermal application	[ 4, 19, 29, 30, 42, 45, 50, 52, 55, 57, 58, 66, 67, 79, 83, 88, 95, 109, 112, 113]	5333	556 (10)	(0–100) [ 30] - [ 19, 45, 83]
Intravesical administration	[ 35, 43, 97]	165	33 (20)	(1–100) [ 35] - [ 43]
Oral administration	[ 9]	15	4 (27)	
**Diarrhea** *(overall incidence)*	[ 2, 18, 41, 54, 57, 85, 93]	1107	27 (2)	(1–6) [ 85] - [ 93]
Intravenous administration	[ 18, 41, 54, 85]	744	15 (2)	(1–6) [ 85] - [ 41]
Transdermal application	[ 2, 57, 93]	363	12 (3)	(2–6) [ 57] - [ 93]

^†^Incidences of the adverse reactions have been calculated for all the individual studies. (min%–max%) are the lowest and highest observed incidence of an adverse reaction observed in the group of studies included. ‡ Nausea and vomiting are reported as one combined adverse reaction in some studies.

**Table 2.** Gastrointestinal adverse reactions observed per number of treatments.

Adverse reaction	Studies	Total treatments, n	Adverse reactions observed, n (%)	(min%–max%) †
**Nausea** *(overall incidence)*	[ 40, 51, 68, 84, 105]	474	161 (34)	(16–57) [ 105] - [ 40]
Intravenous administration	[ 40, 51, 68]	323	137 (42)	(41–57) [ 68] - [ 40]
Intravesical administration	[ 105]	151	24 (16)	
**Vomiting ‡**	[ 51, 68]	316	112 (35)	(29 - 71) [ 68] - [ 51]
**Nausea and/or vomiting ‡**	[ 25, 74, 101]	1557	220 (14)	(8–17) [ 25] - [ 101]
**Abdominal cramps/stomach ache ‡**	[ 51, 68, 101]	495	16 (5)	(1–19) [ 68] - [ 51]
**Halitosis ‡**	[ 68]	262	4 (2)	
**Diarrhea ‡**	[ 51, 101]	233	2 (1)	(1–2) [ 101] - [ 51]

^†^ Incidences of the adverse reactions have been calculated for all the individual studies. (min%–max%) are the lowest and highest observed incidence of an adverse reaction. ‡ Intravenous administration.

The most commonly reported gastrointestinal adverse reactions were nausea and vomiting. The incidence of nausea seems to be less common with the transdermal administration of DMSO compared with intravenous administration. The majority of studies reported an incidence of nausea between 2–14%, with the exception of one study, reporting an incidence of 32% ^2^. In one study that failed to specify the dose, 8 of 42 patients reported nausea and anorexia, but the symptoms disappeared in five of the eight patients when the dose of DMSO was reduced ^45^.

Often the studies had short follow-up periods (less than 24 hours), especially when DMSO was used as a cryoprotectant. The study reporting the highest incidence of nausea had a follow-up period of 5 days ^48^, and the authors concluded that the high incidence of nausea observed might be due to the long follow-up period ^48^. In another article using the same data ^119^, it was suggested that the delayed nausea was due to gastrointestinal mucosal damage, and only the initial nausea could be related to DMSO, and therefore we decided only to include the data from the first 2 days after infusion ^48^.

Halitosis was reported in 29 studies ^4, 9, 16, 19, 29, 30, 35, 42, 43, 45, 50, 52, 55, 57, 58, 66– 68, 79, 83, 85, 88, 94, 95, 97, 98, 109, 112, 113^. In five studies, patients discontinued treatment due to halitosis ^9, 45, 83, 94^. In five studies, all patients experienced halitosis ^9, 45, 83, 94^. Unlike halitosis, other gastrointestinal side effects were reported more often when DMSO was administered intravenously, than transdermally or intravesically.

One study reported a severe case of nausea, vomiting, and abdominal cramps in one patient with an acute allergic reaction ^59^. However, in most studies the reported gastrointestinal reactions were transient and mild, often lasting only minutes to a couple of hours ^16, 38, 41, 68, 85, 87, 90^. Several studies reported a relationship between the dose of DMSO and the occurrence of gastrointestinal adverse reactions ^26, 33, 53, 73, 83, 85^.

### Cardiovascular and respiratory reactions

Cardiovascular and respiratory adverse reactions were reported in 33 studies. Of these, two were randomized controlled trials ^33, 59^, 30 were cohort studies ^7, 18, 23, 25– 27, 36, 39– 41, 51, 54, 61, 65, 66, 68, 73, 74, 80, 85– 87, 90, 100– 102, 104, 115, 117^, and one was a preliminary report ^91^. Except for one study ^66^, all studies reporting cardiovascular and respiratory reactions administered DMSO intravenously ( Table 3 and Table 4).

**Table 3.** Cardiovascular and respiratory adverse reactions observed per number of patients.

Adverse reaction	Studies	Total patients, n	Patients with adverse reactions, n (%)	(min%–max%) †
***Cardiac***				
**Hypotension** ‡	[ 7, 18, 23, 33, 71, 73, 87, 104, 115]	2752	115 (4)	(1–14) [ 18, 71] - [ 87]
**Hypertension** §	[ 7, 18, 23, 33, 41, 54, 61, 73, 85, 87, 102]	2998	385 (13)	(2–95) [ 85] - [ 61]
**Bradycardia (mild and severe)** ‡	[ 23, 36, 54, 61, 65, 85, 90, 91, 115, 117]	882	94 (11)	(0–49) [ 36] - [ 61]
**Decrease in heart rate** ‡	[ 41, 54, 61, 80]	193	152 (79)	(11–94) [ 80] - [ 41]
**Tachycardia** ‡	[ 23, 27, 36]	565	13 (2)	(0–6) [ 36] - [ 23]
**Ventricular extrasystoles** ‡	[ 73]	22	11 (50)	
**Cardiac event, unspecified** ‡	[ 26, 86]	165	18 (11)	(5–12) [ 26] - [ 86]
**Asystole** ¶	[ 91, 100]	45	3 (7)	(3–20) [ 100] - [ 91]
**Left cardiac insufficiency**	[ 85]	194	1 (1)	
**Chest discomfort/tightness** ‡	[ 18, 27, 54, 73, 87, 91, 115]	901	22 (2)	(1–10) [ 27] - [ 54]
***Respiratory***				
**Unspecified respiratory** **symptoms** ‡	[ 26, 86]	165	43 (26)	(21–62) [ 86] - [ 26]
**Dyspnea** ^d^	[ 18, 27, 54, 66, 85]	2748	26 (1)	(0–10) [ 66] - [ 54]
**Cough**	[ 85, 101]	373	52 (14)	(5–22) [ 101] [ 85]
**Lung edema** ‡	[ 59, 85]	241	3 (1)	(1–2) [ 85] - [ 59]

^†^Incidences of the adverse reactions have been calculated for all the individual studies. (min%–max%) are the lowest and highest observed incidence of an adverse reaction. ‡ DMSO was administered intravenously in all studies. § DMSO was administered intravenously in all studies. Horacek *et al.* [ 102] measured 42 patients with an increase in systolic blood pressure, and 31 patients with an increase in diastolic blood pressure. This was counted as 73 cases of hypertension. ¶ In both studies, asystole occurred because of DMSO effect on the trigeminal nerve and activation of the trigeminal cardiac reflex. d) in one study DMSO was administered transdermally

**Table 4.** Cardiovascular and respiratory adverse reactions observed per number of treatments.

Adverse reaction	Studies	Total number of treatments	Adverse reactions observed, n (%)	(min%–max%) †
***Cardiac***				
**Hypotension** ‡	[ 40, 51, 68]	323	10 (3)	(2–14) [ 68] - [ 40]
**Hypertension** ‡	[ 25, 51, 68]	425	60 (14)	(3–21) [ 25] - [ 68]
**Bradycardia (mild and severe)** ‡	[ 51]	54	4 (7)	
**Decrease in heartrate** ‡	[ 39]	32	30 (94)	
**Tachycardia** ‡	[ 51]	54	4 (7)	
**Cardiac event, unspecified** ‡	[ 74]	1269	35 (3)	
**Chest discomfort/tightness** ‡	[ 25, 68, 74]	1640	83 (5)	(0–6) [ 68] - [ 74]
***Respiratory***				
**Dyspnea**	[ 25, 68]	371	3 (1)	(0–2) [ 68] - [ 25]
**Shortness of breath** ‡	[ 74]	1269	40 (3)	

^†^Incidences of the adverse reactions have been calculated for all the individual studies. (%, min-max) are the lowest and highest observed incidence of an adverse reaction observed in the group of studies included. ‡ DMSO was administered intravenously.

Bradycardia was defined as a heart rate less than 60 beats per minute ^41, 61^ and was often transient ^23, 61, 90, 115^, but cases where atropine was needed are described ^49, 96^. A lowered heart rate not enough to be considered bradycardia was observed in four studies ^39, 41, 54, 61^.

In some studies, hypertension did not require intervention ^61, 102^, but cases where medication was needed to control the hypertension, or where treatment was stopped due to hypertension, are described ^41, 54, 85^. Hypotension was also described as transient most of the time ^18, 23, 68, 87, 104^, with some cases needing intervention ^40, 51, 54^.

One study reported 11 cases of transient extrasystoles in 22 patients receiving cryopreserved autologous blood stem cells, monitored with Holter during infusion ^73^. There were two studies reporting cases of asystole during embolization of dural arteriovenous fistulas with a substance called Onyx, a non-adhesive liquid embolic agent dissolved in DMSO ^91, 100^.

Dyspnea was reported in seven studies ^18, 25, 27, 54, 66, 68, 85^. A single study reported eight patients with transient shock after stem cell transfusion ^51^. Some of these patients developed loss of consciousness and cyanosis but recovered promptly and had no need for additional therapy, whereas the rest of the patients developed severe hypotension or transient dyspnea, which was described as the reason for the transient shock. Further description of the condition was not provided.

Several of the studies found a correlation between the dose of DMSO used and the incidence of cardiovascular adverse reactions ^41, 67, 71, 75, 78, 85, 86, 93, 101, 115^.

### Dermatological reactions

Dermatological side effects are common when DMSO is administered transdermally. Skin reactions or allergic reactions were reported in 58 studies. DMSO was applied transdermally in 43 studies ^2, 4, 6, 17, 19– 22, 24, 28– 32, 37, 44, 45, 52, 55, 57, 63, 64, 66, 67, 69, 72, 75, 76, 78, 79, 82, 83, 88, 89, 93, 95, 96, 106, 108, 109, 111– 113^, intravenously in 14 studies ^25, 40, 41, 51, 59, 73, 74, 77, 85, 86, 92, 98, 101, 110^ and intraarticular in one ^103^ ( Table 5).

**Table 5.** Dermatological and allergic adverse reactions observed per number of patients.

Adverse reactions	Studies	Total patients, n	Patients with adverse reactions, n (%)	(%, min-max) †
***Skin reactions***				
**Erythema** ‡	[ 19, 32, 64, 66, 82, 95]	2352	201 (9)	(3–95) [ 95] - [ 82]
**Itching/Pruritus** ‡	[ 6, 55, 57, 64, 66, 72, 82, 93]	3421	215 (6)	(0–70) [ 55] - [ 82]
**Urticaria** ‡	[ 24, 31, 83]	58	9 (16)	(4–59) [ 24] - [ 83]
**Rash**	[ 29, 30, 55, 57, 64, 93, 101, 111]	2682	121 (5)	(1–40) [ 30] - [ 93]
**Paresthesia/burning or** **stinging sensation** § ‡	[ 17, 21, 24, 28, 30, 44, 45, 55, 57, 67, 69, 79, 91, 93, 106]	2141	335 (16)	(0–100) [ 30] - [ 45]
**Scaling of skin/desquamation/** **dry skin/local irritant** ‡	[ 22, 29, 30, 37, 52, 55, 57, 64, 66, 69, 75, 82, 88, 89, 106]	4739	731 (15)	(1–96) [ 66] - [ 52]
**Blistering** ‡	[ 31, 32, 66, 69, 93, 112]	2038	79 (4)	(3–20) [ 66] - [ 112]
**Roughness and/or thickening** **of skin** ‡	[ 66, 82, 93]	1986	191 (10)	(6–10) [ 93] - [ 82]
**Bullous dermatitis/dermatitis** **with vesicles** ‡	[ 20, 29, 64]	1116	79 (7)	(1–9) [ 64] - [ 29]
**Contact dermatitis** ‡	[ 6, 20, 28– 30, 64, 111]	2587	161 (6)	(1–13) [ 28] - [ 29]
**Skin reaction, unspecified** ‡	[ 2, 78, 96, 113]	457	159 (35)	(4–48) [ 96] - [ 113]
**Increase in skin pigmentation** ‡	[ 6]	548	28 (5)	
**Peripheral edema** ‡	[ 45, 55, 66, 109]	2291	22 (0)	(1–14) [ 66] - [ 109]
***Allergic reactions***	[ 37, 44, 59, 86, 98, 110]	309	75 (24)	(3–55) [ 44, 110] - [ 86]
Intravenous administration	[ 59, 86, 98, 110]	229	66 (29)	(2–55) [ 59] - [ 86]
Transdermal application	[ 37, 44]	86	9 (10)	(3–19) [ 44] - [ 37]
Flushing ¶	[ 41, 54, 73]	292	34 (12)	(2–9) [ 54] - [ 73]

^†^Incidences of the adverse reactions have been calculated for all the individual studies. (min%–max%) are the lowest and highest observed incidence of an adverse reaction observed in the group of studies included. ‡ Transdermal application only. § One study administered DMSO through intraarticular injection [ 38]. ¶ DMSO was administered intravenously in all studies.

The most common skin reaction was a local burning sensation reported in 13 studies ^17, 21, 24, 28, 30, 45, 55, 57, 67, 69, 79, 93, 106^. In one study, all participants experienced this burning sensation ^45^. In the same study, four participants experienced a transient peripheral edema associated with itching and erythema ^45^. A single study described a burning sensation in four of 669 patients when DMSO was given as a local injection ^92^; another study described burning in two out of 17 patients when DMSO was injected intraarticularly ^103^.

Most skin reactions were transient, only lasting minutes ^17, 24, 32, 67, 72^, but some studies reported cases described as serious, causing discontinuation of treatment ^2, 6, 52, 63, 78, 96^. There were two studies describing that skin reactions to DMSO would disappear after days of continuous treatment ^45, 83^. Another study reported that 1 of 18 patients treated for psoriasis with DMSO was hospitalized due to exfoliative erythroderma ^63^. In another study, two patients, diagnosed with dermographia developed prominent areas of weals after DMSO application ^95^.

Acute allergic reactions due to use of DMSO were reported in six studies ^37, 44, 59, 86, 98, 110^. One study reported that 63 of 144 patients experienced allergic reactions, which was not described as serious adverse events (bronchospasms, facial flushing, rash) ^86^. In two other studies, acute allergic reactions were characterized as serious adverse events ^59, 110^.

Flushing was regarded as an allergic reaction in this review and was only reported when DMSO was administered intravenously ^25, 40, 41, 51, 54, 73, 74^. A total of four studies, not depicted in Table 5, reported 204 cases of flushing during 1439 stem cell infusions ^25, 40, 51, 74^. Several studies observed a relationship between the dose of DMSO and the occurrence of adverse reactions ^67, 75, 78, 83, 88, 93^.

### Neurological reactions

Headache is the most common neurological adverse reaction reported. In one study, headache was the reason for withdrawal of 2 out of 21 patients being treated with DMSO ^116^.

Three studies using DMSO as a cryoprotectant in stem cell transfusions described seizures after administration ^18, 36, 47^. Severe encephalopathy was observed in one patient ^99^, and transient cranial nerve III and IV palsy was observed in one patient after Onyx embolization ^34^. One study described neurological symptoms occurring during and after transfusion, but they did not define neurological symptoms in detail ^86^.

### Urogenital reactions

Few urogenital reactions were described ( Table 6 and Table 7). Hemoglobinuria was described as an adverse reaction seen after transfusion of stem cell products ^39, 51, 56, 73^. However, hemoglobinuria is often attributed to erythrocyte debris in the transplant material and has thus not been interpreted as being caused by DMSO ^39, 73^. The other urogenital reactions ( Table 6 and Table 7) all occurred after DMSO instillation in the bladder ^38, 49, 97^.

**Table 6.** Neurological and urogenital adverse reactions observed per number of patients.

Adverse reaction	Studies	Total patients, n	Patients with adverse reactions, n (%)	(min%–max%) †
***Neurological***				
**Headache**	[ 2, 18, 29, 33, 38, 41, 55, 59, 70, 71, 81, 84, 85, 98, 101, 104, 116]	2516	150 (6)	(1–50) [ 101] - [ 70]
Intravenous administration	[ 18, 33, 41, 59, 70, 71, 81, 85, 98, 101, 104]	1271	42 (3)	(1–50) [ 101] - [ 70]
Transdermal application	[ 2, 29, 55]	1197	102 (8)	(5–35) [ 55] - [ 2]
Intravesical administration	[ 38]	20	1 (5)	
Rectal administration	[ 116]	21	3 (14)	
>1 administration route	[ 84]	7	2 (29)	
**Seizures**	[ 18, 36, 47]	301	2 (1)	(0–2) [ 18] - [ 47]
**Neurological symptoms,** **unspecified**	[ 86]	144	5 (3)	
**Transient CN III and IV palsy**	[ 34]	12	1 (8)	
**Severe encephalopathy**	[ 99]	124	1 (1)	
***Urogenital***				
**Pelvic discomfort/pain/** **irritation**	[ 38, 49, 97]	107	10 (9)	(6–30) [ 49] - [ 38]
**Dysuria/strangury**	[ 49]	36	6 (17)	
**Renal and urinary disorder**	[ 49]	36	8 (22)	

^†^Incidences of the adverse reactions have been calculated for all the individual studies. (min%–max%) are the lowest and highest observed incidence of an adverse reaction observed in the group of studies included.

**Table 7.** Neurological and urogenital adverse reactions observed per number of treatments.

Adverse reaction	Studies	Total treatments, n	Adverse reactions observed, n (%)	(min%–max%) †
***Neurological***				
**Headache**	[ 39, 51]	86	40 (47)	(6 - 73) [ 39] - [ 51]
***Urogenital***				
**Urethral irritation**	[ 73]	151	110 (73)	

^†^Incidences of the adverse reactions have been calculated for all the individual studies. (%, min-max) are the lowest and highest observed incidence of an adverse reaction observed in the group of studies included.

### Other reactions

Only one study in this review administered DMSO as eye-drops ^114^. In this study, two patients experienced severe conjunctival hyperemia due to allergic reactions, and 25% of patients experienced a stinging sensation when eye-drops were applied ^114^. Other studies performed eye examinations to determine whether DMSO caused changes in the lens; however, no such cases were observed ^2, 45^.

Hyponatremia occurred in six patients after they received large doses of DMSO as treatment for cranial hypertension ^62^. This adverse reaction was not reported in other studies ( Table 8).

**Table 8.** Other adverse reactions observed per number of patients.

Adverse reaction	Studies	Total patients, n	Patients with reaction, n (%)	(min%–max%) †
**Fever**	[ 27, 71, 73, 77, 101]	547	44 (8)	(2–19) [ 27] - [ 77]
**Chills**	[ 27, 33, 70, 71, 81, 85, 101]	852	60 (7)	(1–31) [ 101] - [ 71]
**Dizziness**	[ 2, 46, 55, 85, 101]	885	18 (2)	(1–15) [ 55] - [ 2]
**Weakness**	[ 33, 45, 46]	293	19 (6)	(1–29) [ 46] - [ 45]
**Sedation**	[ 2]	78	34 (44)	
**Hyponatremia**	[ 62]	6	6 (100)	

^†^Incidences of the adverse reactions have been calculated for all the individual studies. (%, min-max) are the lowest and highest observed incidence of an adverse reaction observed in the group of studies included.

Very few cases of serious adverse reactions associated with DMSO have been described ^18, 36, 51, 59^.

Overall, most studies administered DMSO intravenously or transdermally ( Table 9)

**Table 9.** Way of administration of DMSO in included studies.

Administration	Number of studies	References
**Intravenous**	49	[ 7, 16, 18, 23, 25, 26, 33, 34, 36, 39– 41, 46– 48, 51, 53, 54, 56, 59, 61, 62, 65, 68, 70– 74, 77, 80, 81, 84– 87, 90, 91, 94, 98– 102, 104, 110, 115, 117, 118]
**Transdermal**	48	[ 2, 4, 6, 17, 19– 22, 24, 28– 32, 37, 42, 44, 45, 50, 52, 55, 57, 58, 63, 64, 66, 67, 69, 72, 75, 76, 78, 79, 82– 84, 88, 89, 93, 95, 96, 106– 109, 111– 113]
**Intravesical**	7	[ 8, 35, 38, 43, 49, 97, 105]
**Oral**	2	[ 9, 60]
**Eye-drops**	1	[ 114]
**Local injection**	1	[ 92]
**Intra-articular**	1	[ 103]
**Rectal**	1	[ 116]

### Risk of bias within studies

**Figure 2.**
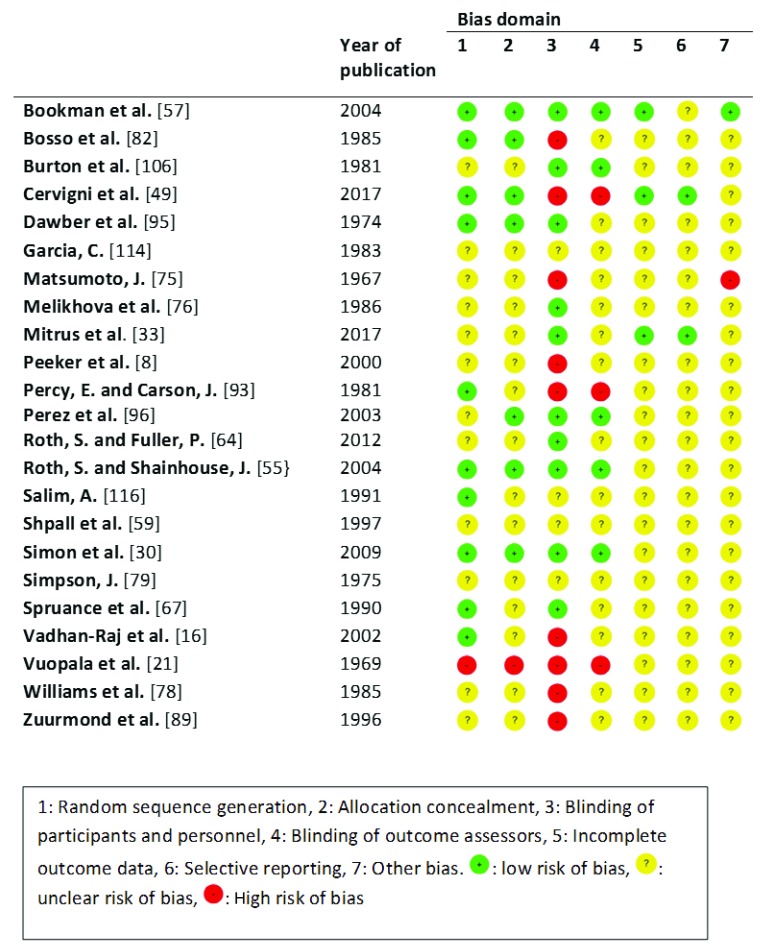
Risk of bias in randomized controlled trials.

In this review, we included 76 cohort studies, of which 64 were prospective ^2, 4, 6, 7, 20, 22, 24– 27, 29, 31, 32, 34– 38, 40– 45, 48, 50– 54, 56, 58, 60, 63, 65, 66, 68– 70, 72, 73, 77, 80, 81, 83, 85, 88, 90, 92, 94, 97, 98, 101– 104, 107, 108, 110, 112, 115, 117, 118^ and 13 were retrospective ^9, 18, 23, 39, 46, 47, 61, 71, 74, 86, 87, 100, 105^. Bias was assessed using The Newcastle-Ottawa-Scale ^14^. Using this scale, studies were given zero to nine stars. A high number of stars equals low risk of bias and vice versa. The studies in this review had a median value of 5 stars, with a range of 2–8. No studies received the highest possible value of nine stars. Very few studies had a comparison group that did not receive DMSO, and often the occurrence of adverse reactions was poorly described. There were 24 randomized controlled trials ( Figure 2). Many studies received an unclear risk of bias because often it was vaguely described how adverse reactions were reported.

Overall, there was a high risk of bias when assessing the description of adverse reactions. Some studies were not assessed for bias due to being case-reports, preliminary trials, or because they included more than one study design ^17, 19, 28, 62, 84, 91, 99, 109, 111, 113^.

## Discussion

Gastrointestinal and dermatological adverse reactions were the most commonly reported in the included studies. Cardiac adverse reactions only occurred when DMSO was administered intravenously, whereas dermatological reactions mostly occurred when DMSO was administered on the skin. Serious neurological and cardiac reactions were rare and only described in few studies. There seems to be a dose-response relationship between DMSO and adverse reactions with no or mild reactions in low doses.

Many studies on the use of DMSO have been performed in Russia. These studies have not been readily accessible to the global community due to the language barrier. In this review, we have included not only studies dating back almost 50 years, but also articles written in Russian, which is an important strength of the review. This study has several limitations: 1) Some studies used the NCI-CTC (National Cancer Institute’s Common Terminology Criteria for adverse events), but often no scale was used, and the occurrence of adverse reactions were poorly reported. 2) It was difficult to make conclusions on the frequency of a specific adverse reaction, because the exact number of patients experiencing a reaction was often not stated. 3) Several studies using DMSO as a cryoprotectant concluded that other factors affected the occurrence of adverse reactions ^7, 85, 86^. One study prospectively looked at the adverse reactions observed in relation to autologous transplantation in 64 European Blood and Marrow Transplant Group centers ^7^. They had difficulties isolating the effects of DMSO from confounding factors such as cell breakdown products and conditioning chemotherapy. Factors such as age, gender, volume transfused, granulocyte concentration, clumping of transplant material, and amount of red blood cells played a role in the occurrence of adverse reactions ^61, 86, 120– 122^. Another study believed that acute volume expansion, electrolyte imbalance and vagal responses to the coldness of the freshly thawed infusate were more likely reasons for cardiac arrhythmias during stem cell transfusions than the DMSO infused ^123^. This differs from other studies, which found a clear connection between dose of DMSO and occurrence of cardiac adverse reactions ^41, 67, 71, 75, 78, 85, 86, 93, 101, 115^. Therefore, it is possible that some adverse reactions are more or less common than found in this review. The rarer side effects are often reported in case reports, which often did not meet the eligibility criteria in this review. However, we have included several larger studies in this review, and they found a very small occurrence of serious adverse events ^7, 55, 66, 74^.

 In conclusion, adverse reactions due to DMSO are often mild and transient and do not qualify as serious adverse events. Cardiovascular and respiratory adverse reactions occur mostly when DMSO is administered intravenously, whereas dermatological reactions have a higher incidence when DMSO is administered transdermally. An important finding is that the occurrence of adverse reactions seems to be related to the dose of DMSO, and it therefore seems safe to continue the use of DMSO in small doses.

## Data availability

All data underlying the results are available as part of the article and no additional source data are required.

## Supplementary material

**Supplementary File 1. Completed PRISMA harms checklist.**
