# Supplementary material for: Adverse reactions of dimethyl sulfoxide in humans: a systematic review
Source: F1000Res. 2019 Aug 6;7:1746. Originally published 2018 Nov 5. [Version 2] doi: 10.12688/f1000research.16642.2 (PMC6707402; doi:10.12688/f1000research.16642.2)
Supplement: Peer review history

# Peer review: Reviewer response for version 2

**DOI:** 10.5256/f1000research.22126.r52126

**Published:** 2019-08-20

**Authors:** Curly Morris

**Affiliations:** 1 Centre for Cancer Research and Cell Biology (CCRCB), Queen's University Belfast, Belfast, UK

The manuscript is now suitable for indexing.

I confirm that I have read this submission and believe that I have an appropriate level of expertise to confirm that it is of an acceptable scientific standard.

# Peer review: Reviewer response for version 2

**DOI:** 10.5256/f1000research.22126.r52125

**Published:** 2019-08-13

**Authors:** Igho J. Onakpoya

**Affiliations:** 1 Centre for Evidence-based Medicine, Nuffield Department of Primary Care Health Sciences, University of Oxford, Oxford, UK

The authors have adequately addressed my concerns.

I confirm that I have read this submission and believe that I have an appropriate level of expertise to confirm that it is of an acceptable scientific standard.

# Peer review: Reviewer response for version 1

**DOI:** 10.5256/f1000research.18188.r45643

**Published:** 2019-03-21

**Authors:** Igho J. Onakpoya

**Affiliations:** 1 Centre for Evidence-based Medicine, Nuffield Department of Primary Care Health Sciences, University of Oxford, Oxford, UK

The authors have conducted a systematic review assessing reports of adverse reactions attributed to DMSO. The topic is interesting, and the authors have conducted their searches in a reasonable way. However, there are several flaws in this manuscript that need to be addressed:

**Introduction**

- The term “possible adverse reactions” is incorrect. Suspected adverse reactions is more reasonable

**Methods**

- If Russian articles were screened by only one author, how were discrepancies resolved in these cases? Specify which authors extracted the data, and whether this was done independently.

**Results**

- The term “possibly due” is incorrect. There are 4 levels in describing associations between medicines and suspected adverse reactions. The authors should revise their terminology.
- You state “in some studies patients discontinued treatments due to halitosis”; however, you have provided references for 5 studies – the report can be more precise.

**Discussion**

- How does “including Russian studies” strengthen the review? What about several other languages that have been omitted?
- You state that there seems to be a dose-response relationship, and have drawn similar conclusions. However, at no point in the results do you report data to support this claim. You state that studies reported associations between dose and the occurrence of adverse reactions, but fail to report the doses in question.
- Please enumerate the limitations of your review.

I confirm that I have read this submission and believe that I have an appropriate level of expertise to confirm that it is of an acceptable scientific standard, however I have significant reservations, as outlined above.

# Response

**Published:** 2019-07-31

**Authors:** bennedikte Madsen

**Affiliations:** Herlev Hospital, Denmark

Dear Igho J. Onakpoya,

 Thank your for reviewing our manuscript “Adverse reactions of dimethyl sulfoxide in humans: a systematic review”. Your comments were very helpful and we appreciate the effort you put in to reviewing our manuscript. We have addressed the individual questions in the section below. We hope your find our replies satisfactory. Questions are written in *italic *and answers in plain.

*Q1:* *The term “possible adverse reactions” is incorrect. Suspected adverse reactions is more reasonable.*

 A1: We have changed the paragraph in the introduction section to “suspected adverse reactions”.

*Q2: If Russian articles were screened by only one author, how were discrepancies resolved in these cases? Specify which authors extracted the data, and whether this was done independently.*

 A1: We have clarified in the manuscript how the screening process was performed *: “*Two authors (B.K.M. and D.Z.) independently screened title and abstract according to the eligibility criteria using www.covidence.org. Discrepancies were resolved by discussion. One author screened the full-text articles (B.K.M.). Russian articles were screened by an author fluent in Russian (M.H.). If M.H was in doubt regarding inclusion of a study the results were presented to B.K.M and then discussed until a mutual decision was made. After the screening process was finished, all included studies were imported to an Excel sheet (Microsoft Excel 2016). Data extraction was performed by two authors (M.H. extracted from the Russian articles and B.K.M. extracted from the rest) *.”*

*Q3: The term “possibly due” is incorrect. There are 4 levels in describing associations between medicines and suspected adverse reactions. The authors should revise their terminology*.

 A3: We have rewritten the paragraph so it now states: “Gastrointestinal adverse reactions were reported in 61 studies. Of these, 10 studies were randomized controlled trials. *”*

*Q4: You state “in some studies patients discontinued treatments due to halitosis”; however, you have provided references for 5 studies – the report can be more precise*

 A4: We have made our report more precise and it now states: “In five studies, patients discontinued treatment due to halitosis.”

*Q5:* *How does “including Russian studies” strengthen the review? What about several other languages that have been omitted*?

 A5: A Russian Chemist, Dr. Alexander Saytzeff, identified DMSO in 1866, however it was not used for medical use at the time ^1^ . But the fact that he was Russian might have been the reason why Russian scientists have made numerous studies using DMSO. We therefore thought it would be valuable to include Russian articles since many of these studies have never been translated, and therefore are not available to the international scientific society. Of course, we could have included many other languages, but we thought it most relevant to include Russian since we observed a large amount of articles in Russian during our initial examination of the subject.

*Q6:  You state that there seems to be a dose-response relationship and have drawn similar conclusions. However, at no point in the results do you report data to support this claim. You state that studies reported associations between dose and the occurrence of adverse reactions but fail to report the doses in question.*

 A6: As mentioned in our study several studies described a dose-response relationship between the amount of DMSO and the occurrence of adverse reactions ( ^26^^,^^33^^,^^41^^,^^53^^,^^67^^,^^71^^,^^73^^,^^75^^,^^78^^,^^83^^,^^85^^,^^86^^,^^93^^,^^101^^,^^115^ ). However, since the doses of DMSO and the route of administration differ between the studies, we were not able to give an exact dose. We can only say that an association seems likely.

*Q7: Please enumerate the limitations of your review*

 A7: We have enumerated the limitations listed in the discussion in the manuscript.

# Peer review: Reviewer response for version 1

**DOI:** 10.5256/f1000research.18188.r40223

**Published:** 2019-02-05

**Authors:** Curly Morris

**Affiliations:** 1 Centre for Cancer Research and Cell Biology (CCRCB), Queen's University Belfast, Belfast, UK

With well over 20,000 patients receiving DMSO based autologous transplants annually in Europe alone, this is a timely review of the toxic effects of this valuable agent. It has been performed in an appropriate and scholarly manner and brings added value by including the Russian literature not easily accessible to the average English-speaking reader.

 However there are ways in which the review might be improved and give added value to the reader.

 It is not easy to ascertain the number of patients receiving DMSO intravenously and those receiving it by other routes. A small table could clarify this. 

 The side effect tables either as numbers of patients or numbers of treatments. If they cannot be presented as one combined set of data then some explanation of the two separate tables would be beneficial.

 There seems to have been no attempt to quantify the dose of DMSO which patients have received or to characterize the severity of the reactions and relate these. Furthermore DMSO is usually a vehicle to facilitate giving the patient some other treatment e.g. a stem cell transplant or drug so the reasons for the use of DMSO are not clear. This also means there are side effects from the drug or treatment facilitated by the DMSO; is it possible to separate these effects in any way? Do the authors of the many papers selected for analysis recommend an upper limit to the amount of DMSO given or have a strategy for minimising the dose?

 In their final paragraph the authors suggest that "reactions due to DMSO are often mild and transient". In their previous paragraph they admit that the case reports the less common and more severe side effects which did not meet the eligibility criteria of this review. However as long ago as 2005 it was possible to identify severe side effects in an appreciable number of cases (Windrum *et al.*, 2005 ^1^). Furthermore although they do not separate the factors responsible the authors of reference 7 record a SAE (Grades 3, 4 and 5) profile in excess of 3%. The authors should possibly be a little more circumspect in this paragraph particularly as they recommend the use of DMSO in (unspecified) small doses.

I confirm that I have read this submission and believe that I have an appropriate level of expertise to confirm that it is of an acceptable scientific standard, however I have significant reservations, as outlined above.

## References

[1] : Variation in dimethyl sulfoxide use in stem cell transplantation: a survey of EBMT centres. *Bone Marrow Transplant*.2005;36(7) : 10.1038/sj.bmt.1705100 601-3 10.1038/sj.bmt.1705100 16044141

# Response

**Published:** 2019-07-31

**Authors:** bennedikte Madsen

**Affiliations:** Herlev Hospital, Denmark

Dear Curly Morris,

 Thank for reviewing our manuscript: “Adverse reactions of dimethyl sulfoxide in humans: a systematic review”. We appreciate the effort put into reviewing our manuscript, and we have tried our best to use your comments to improve our manuscript. We have addressed the individual questions in the section below. We hope your find our replies satisfactory. Questions are written in *italic *and answers in plain.

 Q1: *It is not easy to ascertain the number of patients receiving DMSO intravenously and those receiving it by other routes. A small table could clarify this.*

 A1: We have added a table (Table 9) to our manuscript describing the route of administration of DMSO.

 Q2 & 3: *There seems to have been no attempt to quantify the dose of DMSO which patients have received or to characterize the severity of the reactions and relate these. Furthermore DMSO is usually a vehicle to facilitate giving the patient some other treatment e.g. a stem cell transplant or drug so the reasons for the use of DMSO are not clear. This also means there are side effects from the drug or treatment facilitated by the DMSO; is it possible to separate these effects in any way? Do the authors of the many papers selected for analysis recommend an upper limit to the amount of DMSO given or have a strategy for minimising the dose*?

*In their final paragraph the authors suggest that "reactions due to DMSO are often mild and transient". In their previous paragraph they admit that the case reports the less common and more severe side effects which did not meet the eligibility criteria of this review. However as long ago as 2005 it was possible to identify severe side effects in an appreciable number of cases (Windrum et al., 2005* *^1^* *). Furthermore although they do not separate the factors responsible the authors of reference 7 record a SAE (Grades 3, 4 and 5) profile in excess of 3%. The authors should possibly be a little more circumspect in this paragraph particularly as they recommend the use of DMSO in (unspecified) small doses*.

 A2 & 3: DMSO is most often used as a vehicle in combination with other drugs. Therefore, it is not possible to separate completely the adverse reactions related to the use of DMSO and the adverse reactions related to other drugs, since adverse reactions such as nausea, vomiting, headache etc. are not specific for solely DMSO.

 As described by the authors of reference 7 ^7^, it was difficult to isolate the effect of DMSO from side effects related to conditioning chemotherapy. The only adverse effect that can solely be attributed to DMSO is halitosis. Therefore, we could not conclude that DMSO was the cause of SAE’s in reference 7 and have not included it in our study. Correctly, Windrum et al. ^1^ describes several adverse reactions which may be contributed to DMSO. However, the study does not describe the seriousness of the adverse reactions.

 As described in our study, it is very possible that some events are underrepresented in our study, which is a limitation.

 The upper limit was not described by any studies; on the other hand several studies evaluated different doses of DMSO and found that a lesser amount of DMSO created fewer adverse reactions ( ^61^ ^,^ ^86^ ^,^ ^120^ ^–^ ^122^.). Based on this observation, we feel confident that the use of small amounts of DMSO is recommendable, since DMSO works well as a vehicle. However, limiting the amount would always be desirable.
